# Beyond Chance? The Persistence of Performance in Online Poker

**DOI:** 10.1371/journal.pone.0115479

**Published:** 2015-03-02

**Authors:** Rogier J. D. Potter van Loon, Martijn J. van den Assem, Dennie van Dolder

**Affiliations:** 1 Erasmus School of Economics, Erasmus University Rotterdam, Rotterdam, the Netherlands; 2 Faculty of Economics and Business Administration, VU University Amsterdam, Amsterdam, the Netherlands; 3 Nottingham School of Economics, University of Nottingham, Nottingham, United Kingdom; University of Maribor, SLOVENIA

## Abstract

A major issue in the widespread controversy about the legality of poker and the appropriate taxation of winnings is whether poker should be considered a game of skill or a game of chance. To inform this debate we present an analysis into the role of skill in the performance of online poker players, using a large database with hundreds of millions of player-hand observations from real money ring games at three different stakes levels. We find that players whose earlier profitability was in the top (bottom) deciles perform better (worse) and are substantially more likely to end up in the top (bottom) performance deciles of the following time period. Regression analyses of performance on historical performance and other skill-related proxies provide further evidence for persistence and predictability. Simulations point out that skill dominates chance when performance is measured over 1,500 or more hands of play.

## Introduction

Poker is the most popular card game in the world. Every day, hundreds of thousands of people play poker for real money on the Internet (Online Poker Traffic Reports). In 2013, online poker rooms generated approximately €2.8 billion in gross win (H2 Gambling Capital). The popularity of the game is also evidenced by the many TV reports of major poker tournaments and the number of participants in these tournaments. In 2014, for example, 6,683 people paid $10,000 to participate in the most renowned poker tournament, the Main Event of the World Series of Poker in Las Vegas.

At the same time, there is a widespread controversy about the legality of poker and the appropriate taxation of winnings. A key issue in the debate is whether poker is to be considered a game of chance or a game of skill. Unlike with games of skill, organizing or playing a game of chance is prohibited or restricted in many countries. Also, many countries have a separate gam(bl)ing tax for games of chance, while money won in a game of skill is generally subject to regular income tax. Kelly, Dhar and Verbiest [1] map legislation and case law on poker for various countries, and show that there is great variation. US regulation even differs across states. Over recent years, several law papers have argued that poker is a skill game and should be recognized as such [2–4].

Authorities often have a less permissive stance towards online poker than towards live poker. In the US, for example, the Unlawful Internet Gambling Enforcement Act (UIGEA) that was adopted in 2006 had a major impact: although the act did not forbid online gambling, it did prohibit the transfer of funds to and from online gambling businesses. As depositing money is necessary for playing online poker, this act effectively declared online poker illegal. If poker were to be considered as a skill game and not as gambling, this could be an argument to exempt the online poker business from the UIGEA [4].

Two different research tracks have examined the skill component in poker. One track focuses on developing and calculating measures of skill, and can be traced back to Kadane [5]. Borm and van der Genugten [6], Dreef, Borm and van der Genugten [7–9], and Hendrickx *et al*. [10] propose measures that compare the performances of different types of players, including an informed hypothetical player who knows exactly the cards that will be drawn. The use of their approach is, however, limited to relatively simple games. Because of the virtually infinite number of possible game situations that result from the many different choice (betting) options that players have and because of the importance of players’ hidden higher-order beliefs, the approach cannot be accurately implemented for the most popular form of poker, No Limit Texas Hold’em. Nevertheless, even for simple poker variants, the different studies report a substantial degree of skill. Heubeck [11] reviews the various kinds of proposed skill measures.

The second track of studies takes a more empirically oriented approach. Likewise, these papers suggest that poker involves a skill component. Larkey *et al*. [12] and Cabot and Hannum [2] ran large-scale simulations with different pre-defined playing strategies and find that their more sophisticated strategies perform better. DeDonno and Detterman [13] carried out experiments with student-subjects and demonstrate that the group of players who received strategic instructions during the session outperformed the control group. Siler [14] analyzes online poker data and establishes that performance is related to playing style, and that style and performance differences between players decrease with the level of the stakes.

In the same spirit as some of the analyses in the present paper, Croson, Fishman and Pope [15] and Levitt and Miles [16] examine whether there is persistence in the performance of poker players. Croson, Fishman and Pope analyze how well players who have finished in the top 18 of a high-stakes tournament fare when they are among the final 18 players in a subsequent major tournament, and they compare their results with those from a similar analysis for professional golf. They find that previous finishes predict current finishes, and that the skill differences across the poker players in their sample are similar to those across the golfers. Levitt and Miles analyze a data set that comprises the complete rankings of all players who entered a 2010 World Series of Poker tournament. They report that players who were *a priori* classified as being especially skilled indeed outperformed the other players.

In what follows, we analyze the role of skill in the performance of online poker players, using a large database with 456 million player-hand observations from real money ring games at three different stakes levels. Online poker seems to be the most obvious data source, because the chance-skill debate is especially oriented towards issues regarding the legality of internet poker and the taxation of winnings from online play. Moreover, the vast amount of data that are available allow for powerful analyses.

We define skill as anything that affects a player’s performance other than chance. In a pure game of chance, each player’s expected winnings are zero (in absence of costs) and there normally is no persistence or positive autocorrelation in their performance: players’ performance over a given period is independent of that over any other period. If performance is predictable, the game involves elements of skill.

Our results indicate that skill is an important factor. When we split our sample into subperiods, we find that players whose performance was in the top (bottom) deciles of the previous period perform better (worse) and are more likely to end up in the top (bottom) deciles of the current period. Regression analyses of performance on past performance and other skill proxies reinforce this evidence of persistence in performance.

From a legal viewpoint, the key question is whether skill dominates chance, that is, whether poker is more a game of skill than a game of chance. The answer to this question heavily depends on the duration and intensity of play, as the effect of chance diminishes with the number of hands and eventually cancels out in the long run. (A “hand” is the game that is played between two subsequent shuffles of the deck: dealing of cards, betting, and awarding of the pot.) Our simulations point out that skill predominates after approximately 1,500 hands.

## Data and Descriptive Statistics

For our analyses we use data on real money ring games (“cash games”) played at one of the major online poker sites. We consider No Limit (NL) Texas Hold’em only because this variant is by far the most popular form of poker worldwide. Our data come from an online service called HHDealer. In recent years, several companies have specialized in gathering and trading so-called “hand histories” from online poker rooms. With software applications they continuously collect information on hands played at online poker tables. Many players buy these data to have information on the playing styles of others. Because of limited resources, hand history providers are unable to store data on every hand that is played online. Out of the websites that responded to our inquiries, HHDealer was able to provide the largest number of hands for an uninterrupted period of twelve months. We purchased all available data for the games that had been played at three particular stakes levels in the period October 2009 – September 2010. In poker, stakes levels are distinguished by the size of the small and the big blind bet. To ground our analysis on distinct stakes levels, we selected data from so-called “low”, “medium” and “high stakes” games, with big blind sizes of $0.25, $2 and $10, respectively. For the medium stakes level, the data that we received also contained hands played in September 2009; we treat these as if they were played in October 2009.

The resulting raw data set contains a total of 76.9 million different hands. The average number of players participating in a hand is 5.9, yielding 456.1 million different player-hand observations. Of these, 190.6 million (41.8%) are from the low stakes games, 229.1 million (50.2%) are from the medium stakes, and 36.4 million (8.0%) are from the high stakes. The smallest number of observations recorded in a month was in February 2010 (17.3 million, or 3.8%), and relates to a software change that temporarily made data mining more difficult. The peak was in January 2010 (57.9 million, or 12.7%).

Table 1 summarizes the data. Our sample contains over 600,000 different players (we interpret each account as a separate player). About 457,000 of them played at least one hand at our low stakes level ($0.25 big blind), 230,000 played in the medium stakes game ($2 big blind) and 34,000 played in the high stakes game ($10 big blind). They rarely switched between these three levels: nearly all hands (96%) were played at the stakes level at which the player played most frequently. A minority of players (17%) were active at more than one of the three levels, but even these players still played 90 percent of their hands at their most favorite level.

**Table 1:** Summary Statistics.

		Small stakes	Medium stakes	High stakes	All stakes
Total players		457,063	230,098	33,572	611,484
Total hands (in millions)		190.6	229.1	36.4	456.1
Hands	Mean	417	996	1,085	746
	Minimum	1	1	1	1
	Median	52	82	70	71
	Maximum	341,498	763,791	461,743	764,890
	Stdev	2,648	8,261	7,003	5,909
Big blinds won (raked)	Mean	−39	−51	−20	−49
	Minimum	−13,134	−10,461	−6,886	−13,135
	Median	−19	−29	−21	−25
	Maximum	11,641	26,516	31,348	30,501
	Stdev	203	374	405	307
Big blinds won (not raked)	Mean	0	0	0	0
	Minimum	−8,748	−7,605	−6,438	−8,749
	Median	−9	−21	−19	−15
	Maximum	26,209	44,110	38,631	44,832
	Stdev	255	573	467	433
Big blinds won per 100 hands (raked)	Mean	−99	−104	−106	−104
	Minimum	−21,500	−15,740	−12,673	−20,000
	Median	−28	−31	−24	−30
	Maximum	11,200	15,000	10,030	15,000
	Stdev	494	455	588	461
Big blinds won per 100 hands (not raked)	Mean	−83	−95	−103	−88
	Minimum	−21,000	−15,666	−12,670	−19,400
	Median	−16	−24	−22	−20
	Maximum	11,600	15,000	10,040	15,000
	Stdev	474	451	587	448

The table shows descriptive statistics for our full sample of 456 million player-hand observations from real money ring games No Limit Texas Hold’em at three different stakes levels. For each stakes level and for the three levels combined, the first two rows show the number of players and the number of hands at the aggregate level. The other rows provide player-level statistics on the number of hands played, the total winnings expressed as the number of big blinds won, and the average number of big blinds won per hundred hands. Profitability statistics are shown with and without correction for rake (the commission taken by the operator). Winnings are corrected for rake by adding back rake in proportion to players’ contribution to the pot. The small, medium and high stakes games have big blinds of $0.25, $2 and $10, respectively.

Players who participated in the high stakes game played on average 1,085 hands at that particular level. For the medium and small stakes this number is 996 and 417, respectively. The average number of hands played at the three levels combined is 746. There is much variation across players in the number of hands that they played at the stakes we have selected. One exceptional player was involved in approximately 765,000 hands (0.17% of our sample), while 58.9 percent of all players participated in less than one hundred hands. The degree of concentration is high: the one percent most active players played 58.5 percent of all hands, and 12.0 percent played 90 percent. Most likely, many of the infrequent players were active at other stakes levels than the three included here.

Table 1 also shows statistics on players’ winnings, both before and after the deduction of “rake” (the commission taken by the operator). To compare and combine performance statistics across stakes levels, winnings are scaled by the size of the big blind. For example, a profit of 5 big blinds corresponds to a profit of $50 at the high stakes, and $1.25 at the low stakes. To also account for differences in the number of hands played, performance is expressed as the number of big blinds won per 100 hands (bb/100). For example, a player who has won $20 at a big blind of $2 after playing 400 hands has realized a performance of 2.5 bb/100.

On average, players lost 104 bb/100 after charging of rake. This average win rate is much worse than the ratio of the average total number of big blinds lost (49) and hands played (746), or 6.6 bb/100. The difference is explained by a positive relation between a player’s profitability and the number of hands that she played. This relation may reflect the effect of experience, but can also be a consequence of budget constraints becoming an obstacle after losses.

Rake substantially affects players’ winnings. If a hand is not finished in the first betting round (“pre-flop”), the operator takes a fixed percentage (5% for our data) from the pot with a fixed nominal cap that depends on the number of players at the table. Only 32 percent of all players in our sample achieved a positive overall result after the deduction of rake. In reality, this percentage is slightly higher: players can easily participate in reward schemes and receive deposit bonuses that partly make up for it, and they can enter into so-called “rakeback deals” with affiliates of the operator.

For our analyses of the role of skill in performance we correct for rake, because we do not want our findings to be conditional on the rake structure that is employed by the operator. Rake is not an intrinsic element of the game, and the percentages and caps differ across sites. Moreover, as explained above, the amount of rake that a player effectively pays is not observable.

To correct players’ winnings for rake, we add back the rake in proportion to their contributions to the pot. On average, rake reduces players’ performance by 16 bb/100 in our sample. As a result of the fixed nominal cap, the effect of rake on players’ win rates is larger for games with smaller stakes. In the absence of rake, 37.5 percent of all players would have made a profit. The extreme values for the best and worst win rate in the table were recorded for lucky and unlucky players who played only one or two hands.

## Decile Analyses

Under the null hypothesis that poker is a game of chance alone, there is no relation between a player’s performance scores across different subperiods. Alternatively, if skill plays a material role in the game of poker, we would expect a player’s performance in one particular subperiod to be indicative of her performance in later subperiods. In this section we subdivide players into deciles based on their performance in the first six months of our sample period and examine how the players in these deciles fared in the last six months. In the next section we look at the persistence and predictability of performance through regression analysis.

Our sample period covers twelve consecutive months. We split up our data into the subsamples October 2009 March 2010 and April September 2010, and rank the different players into deciles according to the average number of big blinds they have won per hand across the first period (the “ranking” period). Because small collections of hands are likely to yield very noisy indicators, we filter out players who have played less than 1,000 hands during this ranking period. This leaves a sample of 17,257 players for the small stakes, 16,435 for the medium stakes, and 2,725 for the high stakes. A total of 36,570 players participated in 1,000 or more hands at the three levels combined. On average, they played 5,706 hands each (median: 2,245). Next, we examine the average performance of the various deciles of players over the second period of six months (the “measurement” period). To prevent selection effects, we impose no restriction on the number of hands in this measurement period. As explained in the previous section, hand outcomes are corrected for rake and scaled by the size of the big blind.

Table 2 shows the results for the three individual stakes levels (Panel A, B and C) and for the three levels combined (Panel D). The left part of the table includes the average performance (in bb/100) for each decile over the ranking period (Period 1), while the right part displays how well each decile fared in the measurement period (Period 2). Note that the deciles comprise less players in the measurement period than in the ranking period. Players either ceased to play at some point, moved up or down in stakes, or were simply not covered in our hand histories. For the three stakes levels combined, out of the 36,570 players who played at least 1,000 hands during the first six months, a subgroup of 20,632 were also active during the subsequent six months. On average, the players in this subgroup played 7,038 hands in the first period (median: 2,526) and 4,814 (median: 717) in the second.

**Table 2:** Standard Performance Measure Deciles.

	Period 1				Period 2				
		bb/100				bb/100			
Decile	*N*	un-weighted	weighted by √n	weighted by n	*N*	un-weighted	weighted by √n	weighted by n	Rank
Panel A: Small stakes									
1	1,726	36.6	35.8	34.7	826	−12.9	3.5	6.8	4,034
2	1,725	19.7	19.6	19.5	862	−12.4	4.7	7.5	3,985
3	1,726	13.5	13.5	13.4	866	1.4	5.2	6.7	3,895
4	1,726	9.2	9.2	9.2	842	−2.4	4.7	6.5	3,956
5	1,726	5.9	5.9	5.9	837	−2.3	4.8	6.4	3,925
6	1,725	3.0	3.0	3.0	775	−8.3	3.4	4.9	4,068
7	1,726	−0.1	0.0	0.0	759	−7.5	3.3	5.1	4,141
8	1,726	−4.0	−3.9	−3.8	771	−11.1	1.4	3.8	4,181
9	1,725	−10.2	−10.1	−10.0	790	−26.7	−0.1	4.7	4,320
10	1,726	−30.0	−29.3	−28.4	890	−20.2	−7.5	−1.3	4,587
Correlation						0.370	0.818	0.964	0.074
(*p*-value)						(0.296)	(0.007)	(0.000)	(0.000)
Panel B: Medium stakes									
1	1,644	34.0	33.0	31.6	946	−25.6	−3.5	3.8	4,398
2	1,643	16.0	15.7	15.3	983	−14.7	0.9	6.0	4,229
3	1,644	9.8	9.7	9.6	937	−22.0	1.6	5.9	4,253
4	1,643	6.0	6.0	5.9	923	−14.2	3.0	5.3	3,972
5	1,644	3.0	3.0	3.1	894	−10.2	0.9	3.8	4,132
6	1,643	−0.2	−0.2	0.0	833	−36.2	−2.9	2.3	4,485
7	1,644	−4.4	−4.3	−4.2	821	−18.8	−4.2	1.8	4,627
8	1,643	−9.7	−9.6	−9.5	831	−29.6	−5.8	1.4	4,673
9	1,644	−18.2	−18.2	−18.0	862	−40.5	−10.3	−2.7	4,806
10	1,643	−41.6	−40.8	−39.9	905	−48.8	−18.0	−5.2	5,190
Correlation						0.612	0.733	0.927	0.104
(*p*-value)						(0.066)	(0.021)	(0.000)	(0.000)
Panel C: High stakes									
1	273	36.8	35.7	33.9	177	−4.7	3.5	5.8	783
2	272	16.3	16.1	15.8	171	−12.7	−0.8	2.6	862
3	273	9.7	9.6	9.5	181	4.3	2.8	2.9	797
4	272	5.8	5.8	5.8	191	−0.3	2.2	3.9	793
5	273	2.8	2.8	2.8	182	−3.8	2.1	4.1	817
6	272	−0.1	0.0	0.2	179	−17.0	0.6	3.5	887
7	273	−4.0	−3.9	−3.7	159	−5.6	−1.9	−1.1	832
8	272	−9.1	−9.0	−8.8	148	−7.6	−4.0	−0.8	891
9	273	−16.5	−16.4	−16.1	158	−32.1	−9.5	−4.1	950
10	272	−40.2	−39.2	−38.0	150	−21.5	−9.6	−1.9	906
Correlation						0.636	0.879	0.770	0.088
(*p*-value)						(0.054)	(0.002)	(0.014)	(0.000)
Panel D: All stakes									
1	3,657	34.7	33.8	32.7	2,082	−23.0	−0.2	5.1	10,135
2	3,657	17.5	17.4	17.2	2,126	−13.1	2.2	6.1	9,810
3	3,657	11.3	11.3	11.1	2,140	−12.6	2.7	6.0	9,736
4	3,657	7.3	7.2	7.1	2,105	−6.0	3.8	5.9	9,442
5	3,657	4.1	4.1	4.1	2,099	−13.7	2.3	4.5	9,850
6	3,657	1.1	1.1	1.2	2,004	−13.3	1.3	3.7	9,907
7	3,657	−2.5	−2.5	−2.4	1,939	−16.0	−1.0	3.0	10,382
8	3,657	−7.4	−7.3	−7.3	1,948	−22.0	−3.5	1.7	10,764
9	3,657	−14.9	−14.8	−14.7	2,047	−25.7	−6.0	0.0	11,062
10	3,657	−37.7	−37.0	−36.1	2,142	−42.5	−16.5	−5.9	12,098
Correlation						0.600	0.733	0.927	0.101
(*p*-value)						(0.073)	(0.021)	(0.000)	(0.000)

The table ranks all players who played 1,000 hands or more over the first six months of our sample period (Period 1) into deciles by their performance over these six months, where performance is measured as the average number of big blinds won per hundred hands after correction for rake. For each decile, the first columns show the number of included players (*N*) and their average performance (bb/100) for this ranking period. The next columns show the number of players from the decile who played at least one hand in the last six months of our sample period (Period 2), as well as their average performance for this measurement period and how they rank on average relative to all other players who played at least one hand (Rank). Average decile performance (bb/100) is expressed both as a straight average (unweighted) and as a weighted average, where the weights are either the square roots of players’ number of hands (weighted by √n) or players’ number of hands (weighted by n). Panel A (B/C) shows the results for observations from the small (medium/high) stakes level separately. Panel D shows the results for all stakes levels combined. For each panel, the table shows the Spearman rank correlation between the two periods for the average performance at the decile level (for each weighting method) and for performance at the player level (in Rank column).

In a nutshell, the results in Table 2 indicate that there is substantial and significant persistence in performance: deciles of players that performed relatively well in the first period on average continued to do so in the second period. The findings for individual stakes levels are generally similar to those for the three levels combined, and our discussion below therefore mainly concentrates on the aggregated sample.

We first discuss the results where measurement-period decile performance is calculated as the unweighted average performance across players. In general, players from higher-ranked deciles outperform players from lower-ranked deciles. For example, the average player from the top decile for the three stakes levels combined lost 23.0 bb/100, while the average player from the bottom decile lost 42.5 bb/100; the difference of 19.5 bb/100 is statistically significant (*t* = 3.12; *p* = 0.002). Across all deciles, the Spearman rank correlation between the average decile performances in the ranking period and those in the measurement period is marginally significant (*ρ* = 0.600; *p* = 0.073). At the individual stakes levels, the correlation coefficient is not significantly different from zero for the small stakes, and marginally significant for both the medium and the high stakes.

The unweighted average in period two is negative for all ten deciles. This result is related to the equal weight assigned to every player in calculating decile performance. There is much variation across players in the number of hands they played in Period 2; this number ranges from 1 to 622,936. Because a budget constraint can force a player to stop playing when losses accumulate, a negative average result from a bad sequence of hands is less likely to be cancelled out or diluted by subsequent hands than a positive result after a streak of luck. Consequently, at the player level, negative average performances are more likely to occur than positive average performances. Indeed, players who played relatively few hands in Period 2 have lower scores: those who played less than 100 hands (18.9% of all active players) recorded a score of −79.9 bb/100, while the others (81.1%) recorded −4.6 bb/100 on average.

The substantial share of players who played relatively few hands in the measurement period may also explain why the decile-level correlation between the average performances in the ranking and measurement period is only marginally significant. Performance measurements for infrequent players are relatively noisy, and their widely varying scores consequently distort the strength of the correlation. In fact, players who played only a few hands are given a questionably large weight when decile performance is expressed as a straight average across players. Using a weighted average with players’ number of hands as weights would avoid this problem, and we therefore propose this measure as an alternative indicator. This weighted average is identical to the average profitability per hand across all hands played by the players in a decile combined. Because players who played only infrequently are hardly reflected in this alternative measure, we also consider a compromise weighting method that uses the square roots of players’ numbers of hands as weights.

Evaluating on the basis of the two weighted average performance measures strengthens the pattern observed. Players from higher-ranked deciles again outperform players from lower-ranked deciles in Period 2. For example, hands played by players in the top decile yielded a profit of 5.1 bb/100 across all stakes levels, while hands played by bottom decile players lead to a loss of 5.9 bb/100 (difference: 11.0 bb/100; *t* = 12.36; *p* < 0.001). The Spearman rank correlations across the deciles between performances in the two periods are higher with weighted than with unweighted average scores and always statistically significant – both for the three individual stakes levels and for the three levels combined (for all stakes and weightings: *ρ* ≥ 0.733, *p* ≤ 0.021). Note that the measurement-period performance is positive for most deciles when players’ numbers of hands are used as weights. This is striking, because, by definition, the average winnings per hand are zero across all hands in our unfiltered sample. Apparently, players who played 1,000 or more hands in the prior six months (and thus satisfied our selection criterion) played more profitably than others. This, in itself, might indicate that experience pays off in this game.

The persistence of performance also appears from how players in a given decile rank relative to all other players in Period 2. The last column of Table 2 shows that players from higher-ranked deciles generally rank higher than players from lower-ranked deciles do. For example, for all stakes levels combined, the average rank of top-decile players is 10,135 (out of 20,632), while that of bottom-decile players is 12,098.

At the individual player level, the strength of the correlation between players’ Period 1 and Period 2 ranks is rather moderate. The correlation coefficient ranges between 0.074 (for the small stakes) and 0.104 (for the medium stakes). The relatively low degree of correlation as compared to the correlation coefficients at the decile level reflects the relevance of variance in performance at the individual level—in particular of the variance for players who played only few hands in Period 2. Statistically, however, the rank correlation at the individual player level is highly significant for every (sub)sample (all *p* < 0.001).

As a robustness check, we have also run similar analyses that use three instead of six months as the ranking and measurement period, where we divided our one-year sample period into four non-overlapping quarters (Q1 = October − December 2009, Q2 = January − March 2010, Q3 = April − June 2010, and Q4 = July − September 2010). Regardless of the pair of successive quarters that we use for ranking and measuring, we observe the same pattern of persistence as before: higher-ranked deciles generally outperform lower-ranked deciles. Again, the correlations are stronger when we reduce the influence of relatively infrequent players by calculating performance as a weighted average, and at the individual player level the rank correlation is always highly significant.

Thus far we have ranked players on the basis of their average performance in big blinds. Though simple and natural, this approach ignores the importance of differences between players in the number of hands that they played. Few would share the view that a player who has won 500 big blinds over 1,000 hands (50 bb/100) is to be considered a better performing player than someone who has won 40,000 big blinds over 100,000 hands (40 bb/100). One of the drawbacks of the previous approach is that it does not account for the basic statistical rule that the sampling distribution of the mean depends on the sample size (*n*_*i*_): the greater the number of observations, the less likely that the mean takes an extreme value. For example, if we consider two players with equal ability from a larger population, the player who participates in a smaller number of hands is more likely to be classified in one of the top or bottom deciles if players are ranked by their average winnings per hand. Similarly, the previous approach does not account for differences in playing style or the standard deviation of winnings (*s*_*i*_): when two players are equally profitable, the more adventurous player is more likely to end up in one of the two extremes of the ranking.

We therefore propose an alternative measure to rank players that accounts for the number of hands and playing style of an individual player (*i*):

$$PRM_{i}=\frac{win\;rate_{i}}{stdev\;win\;rate_{i}}=\frac{BB_{i}/n_{i}}{s_{i}/\sqrt{n_{i}}}=\frac{BB_{i}}{s_{i}\sqrt{n_{i}}}$$ (1)

where *BB*_*i*_ is the sum of big blinds won (before deduction of rake), *s*_*i*_ is the standard deviation of big blinds won, and *n*_*i*_ is the number of hands played. We label this measure the “performance robustness measure” (PRM). In fact, *PRM*_*i*_ equals the *t*-value of a test of a player’s observed performance against the null-hypothesis of zero expected performance.

Table 3 presents the new results. Accounting for playing style and number of hands in the ranking period strengthens the previous evidence for performance persistence. Deciles of players who rank higher by their *PRM*_*i*_ generally fare better than lower-ranked deciles. The new ranking method turns out to be more accurate: in many cases, the performance of a decile in Period 2 is now perfectly or almost perfectly monotonically increasing with the rank of a decile for Period 1. For example, for the aggregate data, the rank correlation is perfect when Period 2 decile performance is measured with players’ numbers of hands as weights. For each stakes level, the rank correlation of performance at the individual player level is stronger as well. The new coefficients are about two to four percentage points larger, and range between 0.091 (small stakes) and 0.148 (medium stakes). Additional analyses with three-month periods yielded similar results.

**Table 3:** Performance Robustness Measure Deciles.

	Period 1				Period 2				
		bb/100				bb/100			
Decile	*N*	un-weighted	weighted by √n	weighted by n	*N*	un-weighted	weighted by √n	weighted by n	Rank
Panel A: Small stakes									
1	1,726	26.9	21.1	16.0	908	−1.3	6.2	7.4	3,777
2	1,725	21.0	17.4	13.9	822	1.4	6.1	6.8	3,843
3	1,726	16.1	13.5	10.7	856	−6.0	3.9	5.5	4,008
4	1,726	11.9	10.1	8.2	822	−10.8	3.0	5.4	4,089
5	1,726	7.8	6.7	5.5	819	−8.4	3.7	6.0	4,030
6	1,725	4.1	3.5	2.9	773	−10.9	2.8	5.2	4,094
7	1,726	−0.1	0.0	0.0	784	−7.9	2.6	5.3	4,180
8	1,726	−5.0	−4.4	−3.8	794	−19.0	0.6	3.6	4,236
9	1,725	−11.5	−10.3	−8.8	807	−23.2	0.2	4.6	4,294
10	1,726	−27.6	−24.9	−21.6	833	−17.3	−6.6	−0.6	4,587
Correlation						0.867	0.988	0.939	0.091
(*p*-value)						(0.003)	(0.000)	(0.000)	(0.000)
Panel B: Medium stakes									
1	1,644	20.9	12.6	8.7	1,084	−6.4	4.7	6.0	3,709
2	1,643	19.4	13.7	8.9	946	−8.7	2.0	5.2	4,015
3	1,644	14.4	10.9	7.2	920	−20.5	−0.4	4.3	4,223
4	1,643	9.5	7.7	5.6	867	−23.8	−4.1	2.4	4,600
5	1,644	4.4	3.6	2.6	850	−34.7	−4.2	2.5	4,541
6	1,643	−0.3	−0.2	−0.2	856	−32.7	−5.6	1.1	4,602
7	1,644	−5.6	−4.9	−3.9	827	−26.5	−5.5	2.1	4,670
8	1,643	−11.6	−10.0	−7.9	873	−40.2	−6.5	1.7	4,741
9	1,644	−20.2	−17.7	−14.6	836	−29.1	−8.4	−1.0	4,777
10	1,643	−36.3	−32.1	−26.4	876	−42.6	−14.3	−4.8	5,063
Correlation						0.855	0.988	0.952	0.148
(*p*-value)						(0.004)	(0.000)	(0.000)	(0.000)
Panel C: High stakes									
1	273	24.0	15.1	10.1	204	9.4	4.3	4.1	705
2	272	20.6	14.7	9.1	176	−0.1	2.1	3.6	807
3	273	13.5	9.7	5.9	185	−11.5	0.2	2.1	854
4	272	8.8	6.8	4.6	169	−12.1	−0.6	3.1	863
5	273	4.3	3.3	2.3	173	−5.8	2.0	5.7	832
6	272	−0.1	−0.1	0.0	170	−16.4	0.3	2.1	863
7	273	−5.7	−4.6	−3.6	155	−4.6	−2.6	1.2	868
8	272	−11.1	−9.1	−6.9	159	−8.6	−4.0	−0.7	865
9	273	−18.3	−15.1	−11.3	150	−35.9	−6.5	−2.1	920
10	272	−34.6	−29.1	−22.6	155	−17.9	−10.1	−2.7	959
Correlation						0.697	0.903	0.867	0.118
(*p*-value)						(0.031)	(0.001)	(0.003)	(0.000)
Panel D: All stakes									
1	3,657	23.6	15.6	10.2	2,324	−7.0	5.2	6.2	8,885
2	3,657	19.8	15.1	10.4	2,112	−5.3	3.4	5.3	9,486
3	3,657	14.8	11.7	8.4	2,076	−10.7	1.4	4.8	9,966
4	3,657	10.3	8.4	6.2	2,038	−20.3	0.4	4.4	10,113
5	3,657	6.0	5.0	3.7	2,006	−21.6	0.0	4.2	10,248
6	3,657	1.5	1.3	1.0	2,005	−19.5	−1.4	2.9	10,398
7	3,657	−3.3	−2.8	−2.3	1,970	−23.8	−2.7	2.7	10,654
8	3,657	−9.0	−7.8	−6.3	2,002	−26.1	−4.5	1.5	10,902
9	3,657	−16.7	−14.7	−12.1	2,017	−24.2	−5.1	1.2	10,892
10	3,657	−33.5	−29.8	−24.8	2,082	−31.8	−12.2	−3.9	11,852
Correlation						0.939	1.000	1.000	0.131
(*p*-value)						(0.000)	(0.000)	(0.000)	(0.000)

The table ranks all players who played 1,000 hands or more over the first six months of our sample period into deciles by their performance over these six months. Here, the performance measure that is used to rank players is the performance robustness measure, which is defined as the average number of big blinds won per hand after correction for rake divided by the estimated standard error. The estimated standard error is the sample standard deviation of the rake-corrected winnings per hand divided by the square root of the number of hands. The statistics shown for each resulting decile are defined as in Table 2. The various panels and correlation coefficients are also identically defined.

Another way to look at the persistence of performance is through transition probabilities. Table 4 shows transition probabilities across performance deciles for players who played 1,000 hands or more over the first six months of our sample period. These players are ranked on the basis of their performance twice: for Period 1 and for Period 2. The probabilities in the table indicate the empirical probability of transitioning from a given decile in the first half-year period to a given decile in the second half-year period. Players for whom we have no observations for the second period are not included in the ranking for the second period, so essentially the probabilities are conditional on participation in the second six months.

**Table 4:** Transition Probabilities.

Period 1 decile	Period 2 decile									
	1	2	3	4	5	6	7	8	9	10
Panel A: Ranking by Standard Performance Measure										
1	0.136	0.116	0.098	0.089	0.063	0.078	0.095	0.094	0.108	0.123
2	0.108	0.108	0.131	0.097	0.094	0.086	0.099	0.096	0.089	0.091
3	0.087	0.114	0.124	0.119	0.115	0.099	0.094	0.083	0.079	0.086
4	0.083	0.103	0.124	0.143	0.140	0.103	0.093	0.090	0.069	0.053
5	0.076	0.091	0.118	0.133	0.131	0.126	0.099	0.083	0.071	0.072
6	0.100	0.087	0.093	0.119	0.143	0.119	0.100	0.087	0.079	0.072
7	0.101	0.093	0.094	0.097	0.099	0.121	0.092	0.109	0.104	0.091
8	0.094	0.105	0.079	0.079	0.088	0.109	0.118	0.120	0.105	0.103
9	0.103	0.089	0.081	0.071	0.077	0.097	0.118	0.124	0.130	0.110
10	0.112	0.092	0.056	0.051	0.051	0.065	0.094	0.116	0.166	0.196
Panel B: Ranking by Performance Robustness Measure										
1	0.207	0.133	0.118	0.107	0.091	0.078	0.083	0.067	0.060	0.054
2	0.126	0.117	0.107	0.105	0.108	0.108	0.102	0.091	0.076	0.060
3	0.101	0.110	0.116	0.104	0.106	0.090	0.095	0.104	0.088	0.086
4	0.105	0.104	0.110	0.099	0.093	0.099	0.095	0.112	0.094	0.090
5	0.101	0.090	0.099	0.107	0.104	0.113	0.095	0.100	0.098	0.093
6	0.087	0.092	0.092	0.103	0.112	0.101	0.105	0.102	0.111	0.095
7	0.072	0.108	0.091	0.090	0.099	0.107	0.106	0.102	0.121	0.105
8	0.067	0.089	0.093	0.094	0.106	0.103	0.101	0.111	0.119	0.115
9	0.063	0.078	0.090	0.104	0.100	0.113	0.114	0.100	0.116	0.122
10	0.052	0.074	0.080	0.087	0.081	0.091	0.107	0.116	0.125	0.185

The table shows the transition probabilities across performance deciles for players who played 1,000 hands or more over the first six months of our sample period. Each probability indicates the empirical probability of transitioning from a given performance decile in the first half-year period (Period 1) to a given performance decile in the second half-year period (Period 2). In Panel A, the performance measure that is used to rank players in Period 1 and Period 2 is the standard performance measure, where performance is measured as the average number of big blinds won per hundred hands after correction for rake (bb/100). In Panel B, the performance measure that is used for Period 1 and Period 2 is the performance robustness measure, which is defined as the average number of big blinds won per hand after correction for rake divided by its estimated standard error. Players for whom we have no observations for Period 2 are not included in the Period 2 ranking.

In Panel A, the performance measure that is used to rank players is the standard performance measure (bb/100) after correction for rake. The fraction of players in the top decile of Period 1 who end up in the top decile in Period 2 is 13.6 percent; players who are in the worst decile end up in the worst decile 19.6 percent of the time. These empirical probabilities are substantially greater than the value of 10 percent that would be expected under the null hypothesis of no performance persistence (all *p* < 0.001). At the same time, however, there is some evidence that the likelihood of ending up at the opposite extreme is also greater than 10 percent. For example, the chance of transitioning from the very best (worst) category to the very worst (best) is 12.3 (11.2) percent. This pattern is symptomatic of the inadequacy of the ranking measure used here: players with a higher variance of their average winnings due to adventurous or infrequent play are more likely to end up in the extreme win rate categories. Ranking players on the basis of our alternative performance robustness measure controls for this variance effect.

In Panel B, players are ranked on the basis of their *PRM*_*i*_. The results are compelling: players from the top decile reappear in this decile 20.7 percent of the time, and with a probability of 5.4 percent they end up in the bottom decile relatively infrequently. Similarly, losers are unlikely to become winners: the worst ten percent rank among the best ten percent in the next six months only 5.2 percent of the time and among the worst ten percent 18.5 percent of the time. The empirical probabilities are even more telling when we look at percentiles (not tabulated): the very best one percent of players in Period 1 rank among the very best one percent in Period 2 11.4 percent of the time, and among the best ten percent 32.8 percent of the time (11.4 and 3.3 times the base rate). They are among the worst ten percent only 3.4 percent of the time. Similarly, the least successful players from Period 1 often keep performing poorly: the worst percentile stay in that category 10.2 percent of the time, and belong to the worst decile in 32.0 percent of the cases. They rarely outperform: the best decile is reached only 2.7 percent of the time.

## Regression Analyses

To further analyze the role of skill we regress performance over the final six months of our sample on performance over the first six months, and on other measures that may serve as skill proxies. We consider the following explanatory variables:

- *SPM*: the standard performance measure or “win rate”, defined as the average number of big blinds won per hundred hands after correction for rake.
- *PRM*: the performance robustness measure, defined as the average number of big blinds won per hand after correction for rake divided by the estimated standard error. The estimated standard error is the sample standard deviation of the rake-corrected winnings per hand divided by the square root of the number of hands.
- *Hands (log)*: the natural logarithm of the number of hands played. This variable is a proxy for the experience of players and thus a possible indicator of skill.
- *Tightness*: one minus the proportion of hands in which a player voluntarily wagered money in the first betting round (“called or raised before the flop”). The degree of tightness is one of the two simple measures that are typically used to broadly categorize players’ playing styles. Generally, tighter play is thought to be indicative of a better player. Common mistakes in poker are to impatiently look for “action” and to overestimate the profitability of playing a given hand.
- *Aggressiveness*: the number of times a player led the betting (“bet” or “raised”) as a proportion of the total number of times the player voluntarily wagered money (“bet”, “called” or “raised”). This factor is the other of the two simple playing style measures. Aggressive play is generally thought to yield a higher expected performance than passive play, because increasing the cost of playing at the right times can pressure other players to give up stronger cards or to wager more with weaker ones.
- *Tournaments*: a player’s tournament ability rating according to SharkScope, a website that tracks virtually all online poker tournament results. The worst possible rating is 50 and the best possible rating is 100. The exact calculation is not disclosed by SharkScope. Tournament performance is a possible indicator of skill, because of the many similarities between tournament and cash game play.

The last three variables are standardized such that they have a mean of zero and a standard deviation of one. To avoid endogeneity issues, all six explanatory variables are solely based on data from before Period 2: the first five cover the prior six months (Period 1), and the tournament ability rating is determined over the prior twelve months. The tournament ability rating was available for 79 percent of the players who played 1,000 or more ring game hands in Period 1.

We run two sets of regressions, one for the standard performance measure and the other for our performance robustness measure. In the former case, we face the issue of heteroskedasticity: the variance of the error term is proportional to the sample variance of the number of big blinds won ($s_{i}^{2}$) and inversely proportional to the number of hands played (*n*_*i*_) in Period 2. We therefore apply weighted least squares (WLS) to estimate these regression models, where the weighing factor is the inverse of the variance of the error term ($n_{i}/s_{i}^{2}$). When our performance robustness measure is the dependent variable we use ordinary least squares (OLS), because the errors there have constant variance by construction.

Panel A of Table 5 presents the WLS results for the standard performance measure. In each univariate regression, performance is significantly related to the skill proxy from the previous period (all *p* < 0.001). Not only the historical performance measure (Model 1), but also the number of hands played (Model 2), the two style measures (Models 3 and 4) and the tournament ability variable (Model 5) predict performance to a modest but statistically significant extent. Players who participated in more hands in the previous period perform better, as do players who adopted a tight or aggressive playing style and players who did well in tournaments. Combined, the measures explain 3.3 percent of the variance in performance (Model 6). The smaller-than-unity coefficient in Model 1 indicates that there is regression in players’ performance over time.

**Table 5:** Regression Results.

	Model 1	Model 2	Model 3	Model 4	Model 5	Model 6	Model 7
Panel A: Standard Performance Measure (WLS)							
*Constant*	3.225	−2.536	2.433	3.435	3.984	−0.057	−1.204
	(0.000)	(0.000)	(0.000)	(0.000)	(0.000)	(0.930)	(0.031)
*SPM*	0.167					0.141	0.148
	(0.000)					(0.000)	(0.000)
*Hands (log)*		0.687				0.199	0.338
		(0.000)				(0.004)	(0.000)
*Tightness*			2.048			1.594	1.368
			(0.000)			(0.000)	(0.000)
*Aggressiveness*				1.101		0.668	0.532
				(0.000)		(0.000)	(0.000)
*Tournaments*					0.412	0.509	
					(0.000)	(0.000)	
*R* ^*2*^	0.022	0.007	0.012	0.007	0.001	0.033	0.035
*N*	20,632	20,632	20,632	20,632	16,368	16,368	20,632
Panel B: Performance Robustness Measure (OLS)							
*Constant*	−0.055	−2.267	0.010	0.010	0.009	−1.289	−1.333
	(0.000)	(0.000)	(0.269)	(0.273)	(0.366)	(0.000)	(0.000)
*PRM*	0.229					0.123	0.142
	(0.000)					(0.000)	(0.000)
*Hands (log)*		0.281				0.155	0.161
		(0.000)				(0.000)	(0.000)
*Tightness*			0.242			0.131	0.125
			(0.000)			(0.000)	(0.000)
*Aggressiveness*				0.187		0.080	0.085
				(0.000)		(0.000)	(0.000)
*Tournaments*					0.078	0.041	
					(0.000)	(0.000)	
*R* ^*2*^	0.049	0.049	0.035	0.021	0.004	0.081	0.086
*N*	20,632	20,632	20,632	20,632	16,368	16,368	20,632

The table displays the regression results for our subsample of players who played 1,000 hands or more over the first six months of our sample period (Period 1) and at least 1 hand over the second six months (Period 2). The dependent variable is either the standard performance measure (Panel A) or the performance robustness measure (Panel B). The standard performance measure is defined as the average number of big blinds won per hundred hands after correction for rake (bb/100). The performance robustness measure is the average number of big blinds won after correction for rake divided by its estimated standard error. All explanatory variables are calculated using data from Period 1 only. *SPM* is the player’s standard performance measure. *PRM* is her performance robustness measure. *Hands (log)* is the natural logarithm of the number of hands played. *Tightness* is one minus the proportion of hands in which the player voluntarily wagered money in the first betting round. *Aggressiveness* is the number of times the player led the betting as a proportion of the total number of times she voluntarily wagered money. *Tournaments* is the player’s tournament ability rating from the SharkScope database immediately before the start of Period 2. The results reported in Panel A are weighted least squares regression results with the ratio of a player’s number of hands and her sample variance of the number of big blinds won in Period 2 as weight. Panel B presents ordinary least squares results. The *p*-values are in parentheses.

We obtain qualitatively similar results when we use our performance robustness measure (Panel B), but the explanatory power is higher now. The percentage of variance explained by the joint skill proxies is 8.1 percent, which is about 2.5 times as high as the empirical fit of the previous multivariate specification. We have also performed the regression analyses for the three stakes levels separately. The results and conclusions are all similar to the results for the aggregate sample.

Although these results reinforce our earlier findings of performance persistence and the role of skill in poker, the major part of variance in performance remains unexplained by the models and appears to be attributable to chance. An issue that we have not yet explicitly addressed so far is the problem of errors in variables. In the ideal situation we would know every player’s precise skill level, but given the lack of this information we have to use noisy proxies. When explanatory variables are mismeasured, coefficients estimated via standard regression methods are biased towards zero and the true explanatory power is underestimated. The low empirical fit of the regression models indicates that measurement error is a serious issue for the historical performance measures: if a random factor explains much of the variation in performance, any measurement of previous performance is likely to be subject to a large degree of randomness as well. (Note that the playing style variables are measured with relatively little error because they are based on a large number of draws from a binomial distribution. Their relatively poor predictive power appears to be especially related to their more indirect reflection of skill.)

The bias of an estimated coefficient towards zero as a consequence of measurement error is known as *attenuation* or *regression dilution*. Although measurement error is not a problem for predictive modeling, it can give an unjust impression of the size of the effect of skill on performance here and may falsely suggest that a player’s skill is not a stable quality over time. To account for error in both the dependent and the independent variable we therefore also run a so-called Deming regression [17–19] (methodological details are in S1 Appendix). The results indicate that the standard regression understates the size of the effect of skill on performance to a considerable extent: when we regress the win rate from Period 2 on the win rate from Period 1, we obtain a coefficient of 1.392 (*p* < 0.001). When the performance robustness measure is used for the dependent and for the independent variable, the coefficient is 1.156 (*p* < 0.001). These alternative coefficients are not only substantially higher and closer to unity than the values of 0.167 and 0.229 reported before, but also significantly greater than unity (*p* < 0.001). Taken at face value, this suggests that the disparity in performance between players increases over time. Our robustness analysis at the end of this section, however, indicates that the two coefficients are somewhat inflated as a consequence of players’ budget constraints.

The underestimation of the true explanatory power of skill as a consequence of measurement error decreases with the number of hands used to calculate the proxy for skill. With more observations, measurement error becomes relatively less important: the ratio of the variance of the measurement error and the variance of the true explanatory variable decreases with the number of hands. This holds for each of our two historical performance measures. For the standard performance measure the variance of the measurement error decreases as the number of hands increases. For the performance robustness measure the variance of the measurement error is constant (at unity), but for this measure an increase in the number of hands leads to more distinctive differences between players with a different expected win rate, reducing the relative size of measurement errors.

To illustrate the effect of the number of observations per player on the empirical fit, we run regressions for the pooled results of “teams” of players. More precisely, we first rank players on the basis of their performance in Period 1. We then group the players into percentiles, where the best one percent of players form a group, the second-best one percent form another group, et cetera. Next, for each percentile we calculate Period 1 and Period 2 performance across all hands of the players in the group combined. Last, we regress the pooled Period 2 performance on the pooled Period 1 performance. The average hypothetical “player” has now played about 2.1 million hands in Period 1 (instead of 7,038) and 1.0 million in Period 2 (instead of 4,814). The results are remarkable. When performance is expressed as the win rate the *R* ^2^ is 66.7 percent, and when the performance robustness measure is used the *R* ^2^ is 80.1 percent.

We conclude this section with a robustness analysis. To make sure that the documented persistence of performance truly reflects the role of skill, we need to verify that the results are not driven by differences in budget constraints between players. As already explained in the previous section, a budget constraint can force a player to stop playing when losses accumulate, and, consequently, a negative performance is less likely to be cancelled out or diluted by subsequent hands than a positive performance. The stronger a player’s budget constraint at the start of a given period, the greater the likelihood that she needs to stop early after losses, and the lower her expected SPM and PRM over this period. Differences in budget constraints across players can be both exogenously and endogenously determined: some players may simply have smaller fixed budgets for playing than others in each period, and players who have lost in a previous period have less funds available in their accounts than players who have won. In both cases, the contemporaneous relation between the strength of a budget constraint and performance can lead to spurious correlation in players’ performance through time.

To avoid the possible influence of budget constraints, we use hand samples of a fixed size for every player. For *n* = 1,000, 5,000 and 10,000, we select all players who have played at least 2*n* hands over our entire sample period, and test whether performance over the first *n* hands is predictive of performance over the following *n* hands.

The regression results are in Table 6, and point out that the persistence of performance is robust to this alternative specification. Regardless of *n* and regardless of which of the two performance measures is being used, performance over the second *n* hands is significantly related to performance over the first *n* hands (all *p* < 0.001).

**Table 6:** Regression Results for Fixed Numbers of Hands.

	Model 1	Model 2	Model 3
	(*n*_1_ = *n*_2_ = 1,000)	(*n*_1_ = *n*_2_ = 5,000)	(*n*_1_ = *n*_2_ = 10,000)
Panel A: Standard Performance Measure (WLS)			
*Constant*	2.348	3.656	3.798
	(0.000)	(0.000)	(0.000)
*SPM*	0.066	0.142	0.152
	(0.000)	(0.000)	(0.000)
*R* ^*2*^	0.005	0.021	0.025
*N*	31,991	7,340	3,464
Panel B: Performance Robustness Measure (OLS)			
*Constant*	0.147	0.465	0.705
	(0.000)	(0.000)	(0.000)
*PRM*	0.074	0.136	0.125
	(0.000)	(0.000)	(0.000)
*R* ^*2*^	0.006	0.018	0.016
*N*	31,991	7,340	3,464

The table displays the regression results for subsamples of players who played at least 2*n* hands during our entire sample period, with *n* = 1,000, 5,000 or 10,000. The dependent variable is the player’s performance over the second *n* hands, as measured by either the standard performance measure (Panel A) or the performance robustness measure (Panel B). The explanatory variables are calculated over the first *n* hands. Other definitions are as in Table 5.

There are also two downsides to this alternative approach. First, there is more measurement error because in many cases fewer hands are being used to proxy for skill (especially when *n* = 1,000). Raising *n* solves this issue, but comes at the cost of the inclusion of fewer players. Second, if losing players play less (because of budget constraints or a lost appetite to play), the selection criteria lead to a more homogeneous set of players in terms of their performance. Together with the exclusion of a possible spurious effect of budget constraints, these effects may explain why the *R* ^2^ values for the two present Model 1 specifications (0.5% for SPM and 0.6% for PRM) are remarkably lower than before (2.2% and 4.9%, respectively; see Table 5). Increased measurement error also explains why the regression coefficients are smaller than before. Note that the *R* ^2^ values for the different fixed numbers of hands suggest that decreasing measurement errors are more important than increasing homogeneity when *n* increases from 1,000 to 5,000, and that the two effects are limited or (more or less) cancel out with a further increase to 10,000. Furthermore, the larger regression coefficients for *n* = 5,000 than for *n* = 1,000 again underline the nature and role of measurement error, and the larger constants for larger *n* confirm the selection effect that occurs here.

To account for measurement error we also estimate the six univariate models for the same fixed-size hand samples using Deming regression. Interestingly, all six resulting coefficients are qualitatively close to unity (between 0.86 and 1.11) and only two are statistically significantly different from unity. This result suggests that the Deming coefficients found before were larger than unity due to a spurious effect from budget constraints that is now eliminated, and, more importantly, it points out that skill differences between poker players are close to constant over time.

## Simulations

The previous analyses demonstrate that there is persistence in the performance of poker players. Based on the results, we can confidently rule out that we are dealing with a game of pure chance. Skill is a factor of importance, but the key question left unanswered is whether skill also *dominates* chance, that is, whether poker is more a game of skill than a game of chance. The answer to this question critically depends on the number of hands “the game of poker” is supposed to constitute. The role of chance diminishes with the number of hands, and the law of large numbers implies that it eventually cancels out when the number of hands grows large enough.

In the present section we use simulations to approximate the number of hands above which skill predominates. More specifically, in accordance with the decile analyses, we first rank all players who have played 1,000 hands or more over the first six months of our sample period according to their performance during that first subperiod. Next, for the best performers, we randomly draw (with replacement) a given number of *h* hands from their combined sample of hands recorded for the second six months, and we compute their total winnings (in big blinds) across these *h* hands. We do the same for the worst performers, and then compare the total winnings of the two player categories. For each different value of *h*, we repeat this procedure 25,000 times and determine the proportion of times that the supposedly more skilled players do better than their supposedly less skilled counterparts. To circumvent technical limitations, we draw from a representative subset of one million hands when the actual sample size is greater than one million (we have verified that the results are insensitive to this approximation).

A similar approach was used by Randal D. Heeb in his expert report for a U.S. Federal court case in New York in 2012 (Case 1:11-cr-00414-JBW). For one half of the players in his sample, Heeb estimates a regression model that links performance to hundreds of playing style characteristics, including many different variants of *Tightness* and *Aggressiveness* (see previous section). For the other half, he employs the obtained regression coefficients to compute players’ predicted performance. Heeb’s simulations point out that players who rank high according to this self-constructed skill measure are ahead of lower-ranked players more than 75 percent of the time after only a few hundred hands. A weakness of Heeb’s analysis is that he measures players’ characteristics and their performance over the same set of hands. This is likely to lead to spurious correlation between skill and performance scores, because both scores are contemporaneously co-determined by the same chance elements. For example, players who are dealt a greater fraction of strong hands or hands that connect well with the community cards are more likely to score high on the dimension of aggressiveness (and thus relatively high on skill) and to record a strong performance. Consequently, his analysis is likely to produce an underestimation of the critical number of hands above which skill predominates.

In our simulations, skill predominates if the more skilled players outperform their less skilled counterparts more than 75 percent of the time. This threshold follows from a simple model where we define the skill factor, *π*_*h*_, as the probability that skill determines the more profitable player across *h* different hands. Accordingly, 1 - *π*_*h*_ is the chance factor, or the probability that chance determines the more profitable player. When skill alone determines the winner (*π*_*h*_ = 1), the more skilled player always wins; when chance alone determines the winner (*π*_*h*_ = 0), the more skilled player wins half the time. More generally, the overall probability that the more skilled player is ahead after *h* hands is equal to *p*_*h*_ = *π*_*h*_ · 1 + (1 - *π*_*h*_) · 0.5. Skill predominates when *π*_*h*_ > 0.5, implying *p*_*h*_ > 0.75.

The accuracy of our simulation approach depends on the formation of two distinct groups of players. With each draw of hands, we want to simulate and compare the winnings of a relatively skilled and a relatively unskilled player. Every time, the former is thus assumed to be the better player, with a higher expected performance than the latter. Because we cannot observe a player’s true skill and need to rely on an imperfect proxy, we cannot exclude that our simulations sometimes confuse the two types. To limit this risk, we draw from the hands of players who ended up in either the very best or the very worst performance percentile of the first six months. We use our performance robustness measure to rank players, given our earlier evidence that this measure is more accurate than the standard performance measure. For the sake of completeness, we also run the simulations with the standard performance measure and with the top and bottom deciles of players instead of the top and bottom percentiles.

**Fig 1:**
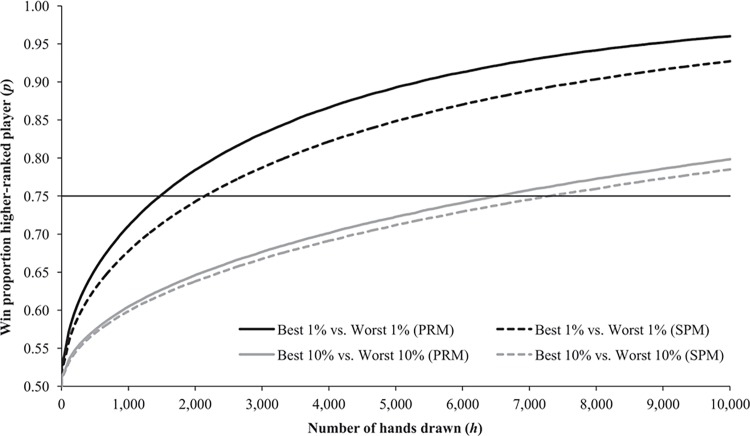
Simulation Results. The figure displays the proportion of times that a selection of *h* randomly drawn hand outcomes for players who were among the best performing players in the past do better than a similar-size selection of hand outcomes for players who were among the worst performing players in the past. Hand outcomes are randomly drawn from the subsample of hands from the second six months of our sample period for players who ranked among the best or worst performing percentiles (black lines) and for players who ranked among the best or worst performing deciles (grey lines) over the first six months of our sample period. Players are ranked according to the performance robustness measure (solid lines) or the standard performance measure (dashed lines). The lines are smoothed, with each point representing the moving average proportion across the simulation outcomes available for *h*-100 up to and including *h*+100.

Fig. 1 displays the results. Across a selection of a few hands, the game is hardly different from a pure game of chance: the higher-ranked players perform better only slightly more than half the time. The proportion steeply increases with the number of hands, at a decreasing marginal rate. (In fact, as *h* increases, the empirical win proportion converges to the win proportion that would result from performance being normally distributed, as predicted by the central limit theorem.) As indicated by the solid black line, the critical point where the best percentile of players (according to the performance robustness measure) is ahead 75 percent of the times is reached after 1,471 hands. As expected, this number is larger when the best and worst percentiles are being selected on the basis of the standard performance measure (2,139), and even larger when deciles instead of percentiles are being used (6,512 and 7,293 for PRM and SPM, respectively).

**Fig 2:**
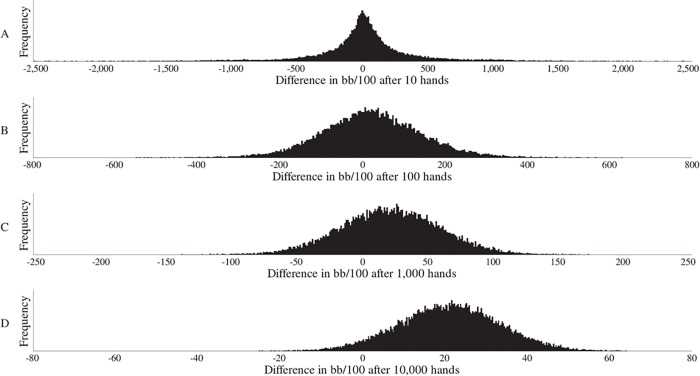
Difference in Win Rate after 10, 100, 1,000 and 10,000 Hands. The histograms A-D display the simulated distributions of the difference in win rate (number of big blinds won per hundred hands) between players who were in the very best and players who were in the very worst performance percentile of the first six months of our sample period. Players are ranked according to the performance robustness measure. For each percentile, *h* hand outcomes are randomly drawn from their subsample of hands from the second six months of our sample period. In A, B, C and D, *h* = 10, 100, 1,000 and 10,000, respectively.

Fig. 2 zooms in on the simulation results for *h* = 10, 100, 1,000 and 10,000, and shows histograms for the distribution of the difference in win rate (number of big blinds won per hundred hands) between the two groups. While the previous figure only shows the proportion of times this difference is positive at a given *h*, the histograms also show the magnitude of the difference in profitability between the higher-ranked and lower-ranked players. Upon visual inspection, the distribution is widely but symmetrically distributed around zero when *h* = 10. The distribution gradually becomes more centered around its mean when *h* increases, and, consequently, with a greater *h* the positive mean win-rate difference of 21.2 bb/100 becomes more apparent (note the different scales for the horizontal axes). At *h* = 10, 100, 1,000 and 10,000, the fractions where the higher-ranked players are behind amount to 47, 43, 29 and 4 percent, respectively. Their chances of underperforming by more than 10 bb/100 shrink from 44 to 39, 21 and 0.5 percent, respectively. At the critical number of approximately 1,500 hands where skill dominates chance, the frequency of underperformance by more than 10 bb/100 amounts to 16 percent.

## Discussion and Conclusions

Our study shows that there is a significant skill factor in online ring game poker, and that this factor dominates the luck factor after a moderate duration of play. In the first sections we have examined whether possible skill differences between online poker players explain differences in their performance. The results in these sections provide strong evidence against the hypothesis that poker is a game of pure chance. For a game of pure chance there would be no correlation in the winnings of players across successive time intervals.

The decile analyses demonstrate that players who rank higher (lower) in profitability over the previous six months generally continue to perform better (worse) than others during the present six months. For example, players from the best decile earn about 20 to 25 big blinds per 100 hands more during the subsequent six months than players from the worst decile. In line with diminishing returns to skill the differences between consecutive deciles are greatest for the ninth and tenth decile. When we rank players on the basis of how well they did according to our alternative performance robustness measure, we find that top ten percent players rank among the top ten percent of the next six months approximately twice as often as others, and among the worst ten percent approximately half as often. The results are even more pronounced if we look at the best one percent. Similarly, those who perform the worst hardly ever end up in the top category.

Our regression results reinforce these findings, and show that current performance is not only related to historical performance but also in some extent to simple measures of playing style. Players who are characterized by a tight or aggressive style generally perform better than their loose or passive opponents. Performance is also related to the number of hands that players have played over the previous period: more frequent or experienced players achieve better results. This finding can indicate that better players choose to play more and that players learn from playing. Both interpretations conflict with the pure-chance hypothesis.

Given these results we believe that we can legitimately conclude that skill is an important factor in online ring game poker. However, most jurisdictions do not ask whether a game involves an *important* degree of skill, but, more specifically, whether skill *predominates*. At the same time, no legislator seems to prescribe how this should be tested. The key complication is that the extent to which skill differences explain differences in performance depends on the number of hands over which performance is measured. If sufficiently many hands are played, skill explains practically all variation in performance. This is nicely illustrated by the high explanatory power of our regressions with the pooled performance scores of percentiles of players, and by the decile analyses, where the (rank) correlation between the past and the current performance of large groups is near-perfect or even perfect. A definite answer to the predominance question thus calls for a definition of the relevant measurement interval. The possible extremes are a single hand and a player’s life time, and intermediate options include an average session, a month, and a (fiscal or calendar) year.

Instead of predetermining one particular interval ourselves, we have employed simulations to estimate the number of hands where skill and chance are equally important. In the previous section we basically run horse races of different durations between a relatively skilled player and a relatively unskilled player who are playing independently from each other. These simulations point out that skill dominates chance when performance is measured over 1,500 or more hands of play. To put this number into perspective: at a rate of 60–80 hands per hour per table, playing 1,500 hands takes people who play only one table at a time about 19 to 25 hours (four to six evenings) of play. Participating on multiple tables simultaneously—which is what many experienced players do—effectively reduces this duration to one or two sessions.

As with any empirical estimate, the exact outcome depends on the specific approach. Our estimate that skill predominates after 1,500 hands should be seen in this light. The simulations do for example not account for possible serial dependence in hand results, which may have led to an underestimation of the critical number of hands. However, we believe that we have taken a conservative approach, because the two types of players in our simulations were not playing the game against each other, and because of selection effects.

With few exceptions, the series of hand outcomes that we compare consist of hands that have been played at tables where the relatively skilled and the relatively unskilled players (virtually) sat down with *other* players than their counterparts in our comparison. The hands were played at different tables and at different moments in time. We are thus not analyzing how well a selection of strong players fare against a selection of weak ones, but comparing how well they did against a cross-section of others, including players from their own category. Would higher-ability players be directly playing against lower-ability players, skill should be expected to predominate substantially quicker.

To avoid extremely noisy historical skill estimates, we have required a minimum data history per player. Unintentionally, this approach is likely to have generated a selection effect: because intensity of play and experience are (almost tautologically) related, relatively inexperienced players are underrepresented in our analyses. Every player in our final sample will normally be well informed about the rules of the game and master some basic strategic concepts. The emphasis on relatively skilled players in our data is probably stronger towards the end of our sample period, because relatively poor players are more likely to lose and quit early, and if they continue to play they have more potential to learn from experience and improve their game. If we would also observe complete beginners playing the game, the differences in performance across players would presumably be greater, and the critical number of hands where skill starts to predominate would consequently be more quickly reached.

The relative homogeneity of our sample is probably strengthened by players’ self-selection into stakes levels on the basis of their perception of their skill level. Better players are more likely to play for larger stakes, while worse or beginning players may feel more comfortable at smaller stakes. This self-selection into the game is not unique for poker. In many games, people play against opponents of relatively similar ability. When such self-selection occurs, the influence of randomness on the outcome of a contest increases, and it will take a longer series of events before skill differences materialize—even with professional sports and with games like chess and bridge.

We conclude with a brief discussion of the generalizability of our findings. Our study has been limited to online play. Due to a lack of readily available data, it is practically impossible to execute an analogous, large-scale analysis for offline play. Nevertheless, given that skill is important in the online variant, we conjecture that it is likely to be even more important for brick-and-mortar play. One reason is that offline play also involves body language and other subtle forms of communication. Players are sitting face-to-face and need to carefully control their behavior to not reveal the strength of their cards, and by observing others they can sometimes discover useful “tells” about their play. At the same time, body language can also be used to deliberately mislead opponents. Furthermore, players’ patience is put to the test more in live play than in online play because fewer hands are dealt per hour. In live poker, skill will probably dominate chance at fewer hands, but because of the slower pace of play and the impossibility to play on multiple tables it may take more hours to reach the critical number.

Another limitation is that we have looked at cash game play only, while both online and offline poker are also frequently played in tournament form. This focus was deliberate, because the value of a given amount of chips wagered in a tournament hand depends on the phase of the tournament and on players’ chip stack size relative to the chip stacks of their opponents. This issue greatly complicates the analysis of performance using hand-level data. A more straightforward approach for tournaments would be to analyze players’ finishes, which is precisely what Croson, Fishman and Pope [15] and Levitt and Miles [16] do for major live events. It is hard to tell whether tournament poker requires more or less skill than the cash game variant, but we believe that a substantial difference is not very likely. In the early phases, tournaments are very similar to cash games, and so will be the roles of chance and skill. At later stages, chance increases in importance because the blind bets become larger relative to players’ chip stacks, which effectively reduces the opportunities for strategic betting. However, for the same reason, meticulous hand selection (which dealt hands to play and which not) then becomes even more consequential. Furthermore, especially at later stages, players also need to factor in the prize money structure in their decisions. Future work could exploit the large amount of available tournament data and see if our speculation that skill similarly predominates after a few sessions of play indeed holds true.

## Supporting Information

S1 Appendix Deming Regression. (PDF)
