# Supplementary material for: Beyond Chance? The Persistence of Performance in Online Poker
Source: PLoS One. 2015 Mar 2;10(3):e0115479. doi: 10.1371/journal.pone.0115479 (PMC4346402; doi:10.1371/journal.pone.0115479)
Supplement: S1 Appendix — (PDF) [file pone.0115479.s001.pdf]

Appendix S1: Deming Regression

Deming regression was first introduced by Adcock (1878). Kummell (1879) extended the method by allowing for the errors in the dependent and in the independent variable to have different variances (although the former was still assumed to be proportional to the latter for all observations). The method is named after the statistician W. Edwards Deming who propagated it (Deming, 1943). For our regression, we use the following model:

(A1) $$y_{i,2}^{*} = \alpha + \varphi\, y_{i,1}^{*}$$

(A2) $$y_{i,t} = y_{i,t}^{*} + \varepsilon_{i,t}$$

where $y_{i,t}^{*}$ are the true values and $y_{i,t}$ are the observed values for the performance of player *i* in period *t* (*t* = 1,2) and where $\varepsilon_{i,t}$ is the measurement error that is normally distributed with a mean of zero.

While standard regression approaches minimize the sum of squares of residuals for the dependent variable ($e_{i,2}$) only, we here minimize the sum of squares of the standardized residuals for both the dependent and the independent variable:

(A3) $$SSR = \sum_{i=1}^{N}\left(\frac{e_{i,2}^{2}}{\mathrm{var}(\varepsilon_{i,2})} + \frac{e_{i,1}^{2}}{\mathrm{var}(\varepsilon_{i,1})}\right)$$

This leads to the following objective function:

(A4) $$\min_{\hat{\alpha},\hat{\varphi},\hat{y}_{1,1}^{*},\cdots,\hat{y}_{N,1}^{*}} \sum_{i=1}^{N}\left[\frac{\left(y_{i,2} - \hat{\alpha} - \hat{\varphi}\,\hat{y}_{i,1}^{*}\right)^{2}}{\mathrm{var}(\varepsilon_{i,2})} + \frac{\left(y_{i,1} - \hat{y}_{i,1}^{*}\right)^{2}}{\mathrm{var}(\varepsilon_{i,1})}\right]$$

As show in York (1966), the solution is:

(A5) $$\hat{\varphi} = \frac{\sum_{i=1}^{N} w_i z_i \tilde{y}_{i,2}}{\sum_{i=1}^{N} w_i z_i \tilde{y}_{i,1}}$$

(A6) $$\hat{\alpha} = \bar{y}_{2}^{w} - \hat{\varphi}\,\bar{y}_{1}^{w}$$

where

(A7) $$\tilde{y}_{i,t} = y_{i,t} - \bar{y}_t^w$$

(A8) $$\bar{y}_t^w = \frac{\sum_{i=1}^{N} w_i y_{i,t}}{\sum_{i=1}^{N} w_i}$$

(A9) $$w_i = \left(v_{i,2} + \hat{\varphi}^2 v_{i,1}\right)^{-1}$$

(A10) $$\mathrm{v_{i,t}} = \mathrm{var}\left(\varepsilon_{i,t}\right)$$

(A11) $$z_i = w_i\left(v_{i,2} y_{i,1} + \hat{\varphi}\, v_{i,1} y_{i,2}\right)$$

In most applications, the variance of the measurement error is not known at the level of individual observations. In order to solve the optimization problem it is then usually assumed that the ratio $\mathrm{var}\left(\varepsilon_{i,2}\right)/\mathrm{var}\left(\varepsilon_{i,1}\right)$ is the same for all *i*. We are in the unique situation that we do have accurate estimates, which allows us to obtain unbiased estimates of φ and α. When the performance of poker players is measured as the average number of big blinds won, the variance of the measurement error var($\varepsilon_{i,t}$) for any specific observation is approximated by $s_{i,t}^2 / n_{i,t}$, or the ratio of the underlying sample variance of the number of big blinds won ($s_{i,t}^2$) and the number of hands ($n_{i,t}$). When our performance robustness measure is used, the measurement error variance is always equal to unity.

References

Adcock, R.J. 1878. “A Problem in Least Squares.” *The Analyst*, 5(2): 53-54.

Deming, W. Edwards. 1943. *Statistical Adjustment of Data*. New York: John Wiley & Sons.

Kummell, Charles H. 1879. “Reduction of Observation Equations Which Contain More Than One Observed Quantity.” *The Analyst*, 6(4): 97-105.

York, Derek. 1966. “Least-Squares Fitting of a Straight Line.” *Canadian Journal of Physics*, 44(5): 1079-1086.
